# Duration of viral infectiousness and correlation with symptoms and diagnostic testing in non-hospitalized adults during acute SARS-CoV-2 infection: A longitudinal cohort study

**DOI:** 10.1016/j.jcv.2023.105420

**Published:** 2023-04

**Authors:** Paul K. Drain, Ronit R. Dalmat, Linhui Hao, Meagan J. Bemer, Elvira Budiawan, Jennifer F. Morton, Renee C. Ireton, Tien-Ying Hsiang, Zarna Marfatia, Roshni Prabhu, Claire Woosley, Adanech Gichamo, Elena Rechkina, Daphne Hamilton, Michalina Montaño, Jason L. Cantera, Alexey S. Ball, Inah Golez, Elise Smith, Alexander L. Greninger, M.Juliana McElrath, Matthew Thompson, Benjamin D. Grant, Allison Meisner, Geoffrey S. Gottlieb, Michael Gale

**Affiliations:** a International Clinical Research Center, Department of Global Health, Schools of Medicine and Public Health, University of Washington, Seattle, WA, United States; b Department of Epidemiology, School of Public Health, University of Washington, Seattle, WA, United States; c Division of Allergy and Infectious Diseases, Department of Medicine, School of Medicine, University of Washington, Seattle, WA, United States; d Department of Immunology, Center for Innate Immunity and Immune Disease, University of Washington, Seattle, WA, United States; e Center for Emerging & *Re*-emerging Infectious Diseases, University of Washington, Seattle, WA, United States; f Global Health Labs, Bellevue, WA, United States; g Department of Laboratory Medicine and Pathology, University of Washington, Seattle, WA, United States; h Vaccine and Infectious Diseases Division, Fred Hutchinson Cancer Research Center, Seattle, WA, United States; i Department of Family Medicine, School of Medicine, University of Washington, Seattle, WA, United States; j Department of Global Health, Schools of Medicine and Public Health, University of Washington, Seattle, WA, United States; k Environmental Health & Safety Department, University of Washington, Seattle, WA, United States

**Keywords:** COVID-19, SARS-CoV-2, Infectiousness, Isolation, Transmission

## Abstract

**Background:**

Guidelines for SARS-CoV-2 have relied on limited data on duration of viral infectiousness and correlation with COVID-19 symptoms and diagnostic testing.

**Methods:**

We enrolled ambulatory adults with acute SARS-CoV-2 infection and performed serial measurements of COVID-19 symptoms, nasal swab viral RNA, nucleocapsid (N) and spike (S) antigens, and replication-competent SARS-CoV-2 by viral growth in culture. We determined average time from symptom onset to a first negative test result and estimated risk of infectiousness, as defined by positive viral growth in culture.

**Results:**

Among 95 adults, median [interquartile range] time from symptom onset to first negative test result was 9 [5] days, 13 [6] days, 11 [4] days, and >19 days for S antigen, N antigen, culture growth, and viral RNA by RT-PCR, respectively. Beyond two weeks, virus growth and N antigen titers were rarely positive, while viral RNA remained detectable among half (26/51) of participants tested 21–30 days after symptom onset. Between 6–10 days from symptom onset, N antigen was strongly associated with culture positivity (relative risk=7.61, 95% CI: 3.01–19.22), whereas neither viral RNA nor symptoms were associated with culture positivity. During the 14 days following symptom onset, the presence of N antigen remained strongly associated (adjusted relative risk=7.66, 95% CI: 3.96–14.82) with culture positivity, regardless of COVID-19 symptoms.

**Conclusions:**

Most adults have replication-competent SARS-CoV-2 for 10–14 after symptom onset. N antigen testing is a strong predictor of viral infectiousness and may be a more suitable biomarker, rather than absence of symptoms or viral RNA, to discontinue isolation within two weeks from symptom onset.

## 1 Introduction

Over 600 million cases of confirmed severe acute respiratory syndrome coronavirus-2 (SARS-CoV-2) infections and 6.5 million deaths from coronavirus disease (COVID-19) have been reported to the World Health Organization (WHO), [1] and these numbers may be underestimated. [2,3] Implementation of diagnostic testing for acute SARS-CoV-2 infection has been critical to identify COVID-19 cases, reduce transmission, and inform public health measures. [4] Testing for SARS-CoV-2 has relied on laboratory-based molecular testing, especially reverse transcriptase polymerase chain reaction (RT-PCR), [5] but the emergence of rapid diagnostic tests has expanded equitable access to diagnostic testing worldwide. [6], [7], [8]

While antigen-based and nucleic acid amplification tests (NAATs) can diagnose SARS-CoV-2 infection and COVID-19 disease, [9], [10], [11] the indication and interpretation of these tests differ. [8] Rapid antigen-based tests are considered less sensitive than NAATs, [12,13] and have not been universally endorsed. [14] However, rapid antigen-based tests have now become widely abundant in community settings [15] and may be useful to facilitate testing and inform isolation policies. [[8], [16], [17], [18]]

The presence of replication-competent virus, as measured by in vitro viral growth, can serve as an imperfect proxy for individual infectivity or contagiousness, [19], [20], [21], [22] but, given the technical and biosafety resources required, is not feasible for routine testing. [23,24] As COVID-19 diagnostics and outpatient treatments become more accessible, there is a growing need to understand the duration of viral infectiousness and correlations with COVID-19 symptoms and diagnostic tests for acute, non-severe SARS-CoV-2 infection. [25]

Current public health guidance offer a range of durations of isolation, from 5 to more than 20 days, for SARS-CoV-2-infected individuals to help reduce viral transmission. [26], [27], [28] These recommendations depend on a person's vaccination status, ongoing symptoms, and serial testing, but are inconsistent and informed by sparse data. Therefore, we characterized the kinetics and variations of viral RNA, viral antigens, and replication-competent virus, including isolation and viral growth assessment of several variants of interest/concern (VOI/VOC), during and after an acute SARS-CoV-2 infection to determine the duration of viral infectiousness with replication-competent virus, and predictors of ongoing individual infectiousness among COVID-19 symptoms and diagnostic tests for acute SARS-CoV-2 infection.

## 2 Methods

### 2.1 Study design and participants

We conducted a prospective cohort study with scheduled serial measurements among adults who had their first SARS-CoV-2 infection from November 2020 to November 2021. Eligible participants were age >=18 years, had no known prior SARS-CoV-2 infection, and had not received COVID-19 vaccination. All SARS-CoV-2 infections were confirmed by RT-PCR from a nasal or nasopharyngeal swab within seven days of enrollment and participants did not require hospitalization. We excluded persons who were pregnant, had an immunological-altering condition (e.g., HIV, Type 1 diabetes mellitus, multiple sclerosis, lupus, and rheumatoid arthritis), were receiving immune-altering medications (e.g., glucocorticoids or immunomodulators), had received treatment for SARS-CoV-2 or associated infections, or were enrolled in an interventional COVID-19 clinical trial. The institutional review board at the University of Washington (STUDY00009981) approved the study.

### 2.2 Procedures

Participants completed standardized questionnaires with comprehensive data, including onset and duration of COVID-19 symptoms. [29,30] After enrollment, participants were scheduled for five additional clinical follow-up visits with pre-defined windows. During each clinical encounter, medical assistants obtained an anterior nasal (AN) swab (Puritan™ PurFlock™ Ultra Sterile Flocked Swabs 253,806 U), a nasopharyngeal (NP) swab (VWR Flocked Nasopharyngeal Specimen Swabs 97–2012), and venous blood. The NP swab was placed in Teknova Viral Transport Medium (VTM) for SARS-CoV-2 and influenza A and B testing using RT-PCR on a Panther Fusion System (Hologic, Inc, Marlborough, USA). Residual VTM was stored at −80⁰ C for viral cultures.

We isolated SARS-CoV-2 and assessed viral growth regardless of RT-PCR result. We prepared two viral growth assays per sample using Vero E6 cells expressing human angiotensin-converting enzyme 2 and transmembrane Serine Protease 2 (VeroE6AT cells). We used microscopy to evaluate cultures for syncytia formation and/or cellular death for 10 days. For virus-positive cultures, we quantified virus titer as a median tissue culture infectious dose (TCID_50_) value using 10-fold serial dilutions. We performed whole genome viral sequencing from the culture isolates on an Illumina NextSeq 500 (Illumina, San Diego, USA), along with positive and negative controls. Sequence reads were processed, de-multiplexed, and assembled against the SARS-CoV-2 Wuhan-Hu-1 ancestral reference genome (NC_045512.2). For each genome, >1 million raw reads were acquired, representing >750x mean genome coverage and a minimum of 10x base coverage. Each consensus genome was analyzed and assigned a lineage based on Phylogenetic Assignment of Named Global Outbreak Lineages (Pangolin) nomenclature, initially in August 2021 (v3.1.11), and again in February 2023 using the latest available release (v4.2). [31]

We tested AN swabs for nucleocapsid (N) and spike (S) antigens using an electrochemiluminescence immunoassay. [32,33] Dry AN swabs were resuspended in 500 µL VTM, [34] incubated for 10 min at room temperature, and lysed with the addition of 1% Igepal CA-630. We added a heterophilic blocking reagent (Scantibodies, Santee, USA) to a concentration of 1.5 mg/mL to prevent non-specific binding. The N antigen assay used antibody pairs 40,143-MM08 and 40,143-MM05 (Sino Biological, Wayne, USA). [32,35] The S antigen assay used antibody pairs 447 (AbCellera Biologics Inc., Vancouver, Canada) and 40,591-MM43 (Sino Biological, Wayne, USA). [36] Plates were read on a MESO QuickPlex SQ 120 plate reader (MesoScale Diagnostics, Rockville, USA) for quantitative concentrations. We fitted a four-parameter logistic function and calculated limits of detection (LOD).

We tested serum samples for SARS-CoV-2 total (IgG +IgM +IgA) anti-spike antibody titers using the Roche Cobas e411 system (Roche Molecular Diagnostics, Indianapolis, USA), and for SARS-CoV-2 anti-spike IgG antibody titers using a chemiluminescent microparticle immunoassay (Abbott Architect SARS-CoV-2 IgG II assay) on the AdviseDx platform (Abbott Diagnostics, Chicago, USA). The total anti-S antibody titers were reported as units per mL (U/mL), which were equivalent to a universal measurement of binding antibody units (BAU) per mL. [37] The anti-S IgG antibody assay provides quantitative results with an “index value” being the ratio of the chemiluminescent signal between the test:calibration samples. Results were provided as arbitrary units per mL (AU/mL), which were then converted to binding antibody units (BAU) per mL [antibody(BAU/mL)=antibody(AU/mL)/7], as indicated by the manufacturer. [37]

### 2.3 Statistical analyses

We defined onset of symptoms as the day any COVID-19 symptom was reported by the participant. We calculated viral load (copies/mL) from RT-PCR using a standard curve that associated Ct value of the Orf1 gene to known viral quantity measured via serial dilutions of AcroMetrix Custom SARS-2 (COVID-19) Full Process Viral controls (R^2^=0.9963). Infectiousness was defined as presence of any replication-competent SARS-CoV-2 in viral cultures, regardless of TCID_50_ value. Analyses of immunological responses were performed both including and excluding results from specimens collected after a COVID-19 vaccination that some participants received during the follow-up period. All analyses were conducted using R.

Robust Poisson regression models were generated to estimate the relative risk of culture positivity for each diagnostic test. We stratified each model by the presence of a set of symptoms (three iterations of each test model): loss of taste/smell, fever, and respiratory symptoms. Additionally, we stratified results by categorical days since symptom onset (0–5, 6–10, 11–14 days), and limited analyses to visits with complete diagnostic testing. We performed separate analyses to estimate relative risk of culture positivity both overall and among people with symptoms, when adjusted for age, sex at birth, comorbidities, and variant.

We used LOESS to fit a smooth curve through the quantitative data corresponding to each testing modality by days from symptom onset. We set values to one-half of the lower limit of quantitation or doubled the upper limit of quantitation for plotting testing results below and above the limits of quantitation, respectively. Median time from symptom onset to a first negative test result for viral antigen, replication-competent virus, and viral RNA was calculated among the individuals with a negative diagnostic test result during visits.

## 3 Results

Among 106 recruited adults, four were ineligible and seven were excluded (Suppl Fig. 1). Among the 95 participants included, median age was 29 years, 43% were female, and most (67%) reported a known SARS-CoV-2 exposure (Table 1). Median time from symptom onset to day of enrollment was six days (Suppl Table 1). During the follow-up period, 30 (32%) participants received a COVID-19 vaccination. All participants tested negative for Influenza A and B.

**Fig. 1:**
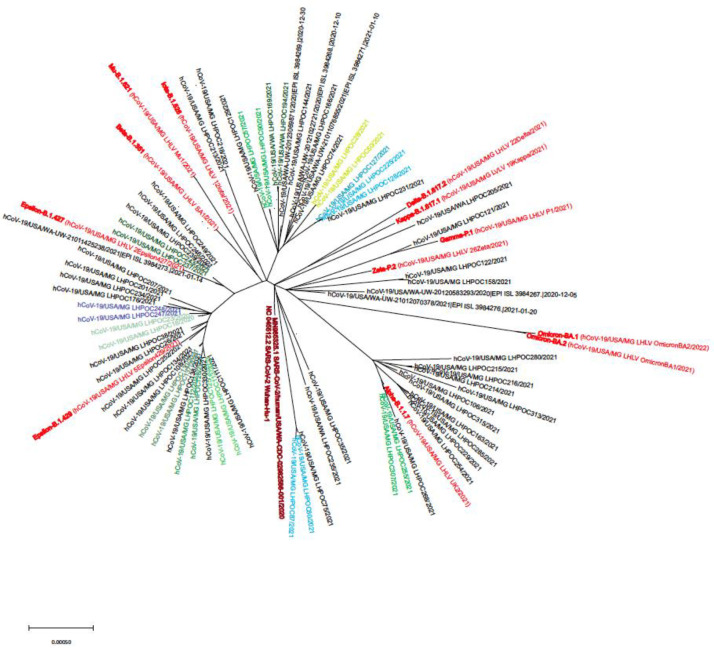
Viral phylogram for unvaccinated adults presenting with acute SARS-CoV-2 infection. Phylogram on the SARS-CoV-2 virus and variants among the enrolled participants (red font indicates reference strains; similar colors indicate related samples, either household contacts or samples from the same individual at different time points).

**Table 1:** Characteristics of the study participants (*N* = 95).

	N (%)^a^
Age (years); median [IQR]	29 [24,38]
Female sex assigned at birth	41 (43)
Race and ethnicity^b^	
American Indian or Alaskan Native	4 (4)
Native Hawaiian or Pacific Islander	1 (1)
Hispanic or Latinx	18 (19)
Asian	11 (12)
White	65 (68)
Black or African American	8 (8)
Other race or ethnicity	1 (1)
Comorbidities	
Any chronic health condition	16 (17)
Diabetes (Type II)	2 (2)
Hypertension	5 (5)
Other chronic health conditions^c^	11 (12)
BMI (kg/m^2^); median [IQR]	25.8 [22.9, 31.4]
SARS-CoV-2 exposure	
Yes, known exposure	64 (67)
If yes, exposure was within household	46 (48)
No known exposure or don't know	31 (33)
Symptoms at initial RT-PCR test date	84 (88)
Days between positive RT-PCR test and enrollment; median [IQR]	4 [3,6]
Days between symptom onset and enrollment; median [IQR]	6 [5,8]
COVID-19-like symptoms in two weeks prior to enrollment	95 (100)
Fatigue	80 (84)
Cough	70 (74)
Aches or muscle pains	69 (73)
Headache	69 (73)
Chills	68 (72)
Runny nose	60 (63)
Loss of taste	53 (56)
Loss of smell	53 (56)
Sore throat	49 (52)
Fever	46 (48)
Shortness of breath/difficulty breathing	43 (45)
Diarrhea	38 (40)
Nausea	33 (35)
Vomiting	10 (11)
Presence of symptoms at visit (n/N (%))	
Visit 4 (∼14 days after enrollment)	36/82 (44)
Visit 5 (∼28 days after enrollment)	3/79 (4)^d^
Visit 6 (∼56 days after enrollment)	5/79 (6)^e^
COVID-19 Vaccination Status	
Vaccinated within 1 month of enrollment	8 (8)
Vaccinated within 2 months of enrollment	22 (23)
Vaccinated during study period	30 (32)
SARS-CoV-2 Variant	
Alpha	9 (9)
Epsilon	22 (23)
Gamma	1 (1)
Other (non-VOI/VOC)	30 (32)
Not sequenced	33 (35)

BMI=body mass index; IQR=interquartile range; VOI=variant of interest; VOC=variant of concern.

a Percentages may not add to 100 due to rounding.

b Percentages for race and ethnicity add to more than 100 because participants could select more than one response.

c Other included lung conditions (*n* = 5, 2 with asthma and 1 with COPD), hypothyroidism (*n* = 1), and not otherwise specified (*n* = 5). No participants reported other chronic heart, kidney, or liver conditions.

d 3 of 3 participants reporting symptoms at V5 reported symptoms at V4; 0 of 3 did not attend V4.

e 1 of 5 participants reporting symptoms at V6 reported symptoms at V5; 1 of 5 did not attend V5. Of the 3 participants who were symptomatic at V6 and asymptomatic at V5, 3 reported symptoms at V4. At V6, the symptoms reported by these 3 participants were loss of sense of taste and loss of sense of smell.

Sixty participants had a sequenced viral variant that was identified using Pango lineage designations at time of infection and corresponding CDC criteria for VOI/VOC: 32 VOI/VOC [9 alpha (B.1.1.7), 22 epsilon (B.1.427, B.1.429), 1 gamma (P.1.17)] and 30 non-VOI/VOC virus (Table 1). Two additional participants had genetically related viral isolates that were initially designated as being of an unknown variant (Pangolin v3.1.11; August 2021) but later updated to lineage B.1.637.1, a second generation variant (non-VOI/VOC), during a reanalysis of the sequencing data with updated pangolin nomenclature (v4.2; February 2023). Phylogenetic analyses indicated a diversity of viral lineages compared to known reference strains (Fig. 1). In stratified analyses, there were no major differences in cohort characteristics or disease presentation by variant (data not shown).

**Fig. 2:**
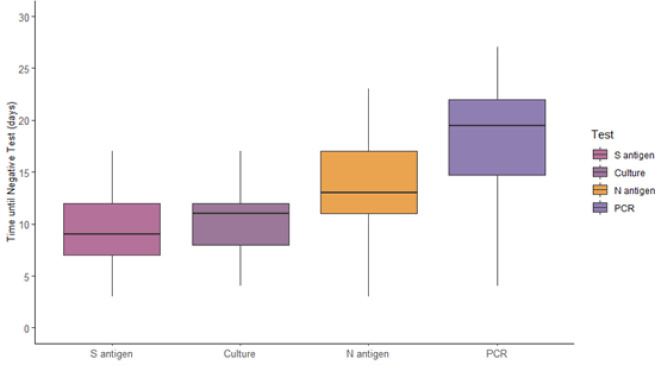
Median days from symptom onset to first negative test among spike (S) antigen, viral culture, nucleocapsid (N) antigen, and RT-PCR for viral RNA. Median [interquartile range] days from symptom onset to the first negative test was 9 [5] days for S antigen, 11 [4] days for viral culture, 13 [6] days for N antigen, and >19 days for RT-PCR, among participants testing negative within 14 days of enrollment. Median for RT-PCR could not be precisely approximated because more than half of participants were positive at all available sample times (within 14 days of enrollment, which corresponds to 2–4 weeks after onset of symptoms).

When categorizing participants by RT-PCR, viral culture, and N antigen positivity, the vast majority (80%) were positive by all three measures during the first 5 days from symptom onset (Table 2). Between 6–10 days from symptom onset, most (96%; 68/71) samples were positive by RT-PCR, 79% (56/71) were N antigen-positive, and 41% (29/71) were culture-positive. Between 11–15 days from symptom onset, 8% (8/96) tests were culture positive and 6% (6/96) were negative by all three tests. Most were positive by RT-PCR and negative by culture (85%; 82/96). Of these 82 tests, 39% were antigen positive and 61% were antigen negative. Beyond 15 days, viral cultures and N antigen titers were rarely positive. Conversely, the RT-PCR test remained positive in 60% (62/104) of participants between 16–30 days after onset of symptoms. Among those, 51% (26/51) of participants remained positive by RT-PCR test between 21–30 days after symptom onset. Overall, the estimated median [interquartile range] time from symptom onset to first negative test result was 9 [5] days, 11 [4] days, 13 [6] days, and >19 days for S antigen, viral culture growth, N antigen, and viral RNA by RT-PCR, respectively (Fig. 2, Suppl Table 2).

**Table 2:** Diagnostic test kinetics of RT-PCR, culture, and nucleocapsid (N) antigen positivity, categorized by days since symptom onset.

Measure				Days since symptom onset^a^						Total
RT-PCR	Culture	N antigen	—	0–5 % (n)	6–10 % (n)	11–15 % (n)	16–20 % (n)	21–25 % (n)	26–30 % (n)	% (N)
+	+	+	*All positive*	80 (28)	41 (29)	1 (1)	0 (0)	0 (0)	0 (0)	19 (58)
+	+	–	*RT-PCR/culture positive*	3 (1)	0 (0)	5 (5)	4 (2)	0 (0)	8 (1)	3 (9)
+	–	+	*RT-PCR/antigen positive*	6 (2)	37 (26)	33 (32)	6 (3)	3 (1)	0 (0)	21 (64)
+	–	–	*Only RT-PCR positive*	3 (1)	18 (13)	52 (50)	58 (31)	46 (18)	50 (6)	39 (119)
–	+	+	*Culture/antigen positive*	0 (0)	0 (0)	0 (0)	0 (0)	0 (0)	0 (0)	0 (0)
–	+	–	*Only culture positive*	0 (0)	0 (0)	2 (2)	0 (0)	0 (0)	0 (0)	1 (2)
–	–	+	*Only antigen positive*	0 (0)	1 (1)	0 (0)	2 (1)	3 (1)	0 (0)	1 (3)
–	–	–	*All negative*	9 (3)	3 (2)	6 (6)	30 (16)	49 (19)	42 (5)	17 (51)
			Column total	35	71	96	53	39	12	306

This table presents data from 306 participant visits–by 95 unique participants–with a complete set of three test results, within 30 days following symptom onset.

“Measure” column indicates the set of three test results collected at the same participant visit.

a Color shading indicates the relative percentage of people within the 5-day periods of days since symptom onset, ranging from dark green (≥80%) to no shading (0%). Most samples (80%) collected at a visit within 5 days of symptom onset were positive by all measures. Agreement quickly waned: samples with culture negative results were frequent 6–15 days after symptom onset. PCR positivity was maintained for many samples collected at visits well beyond 10 days from symptom onset.

We observed a few instances (*n* = 4) of apparent viral “rebound” where Ct values were above Ct >30 but then declined to ≤30 on a subsequent test (Suppl Fig. 3A). In two occurrences the rebound was documented from an RT-PCR test collected more than ten days after symptom onset. However, during the same visit, culture of the residual VTM and an N antigen test were both negative.

**Fig. 3:**
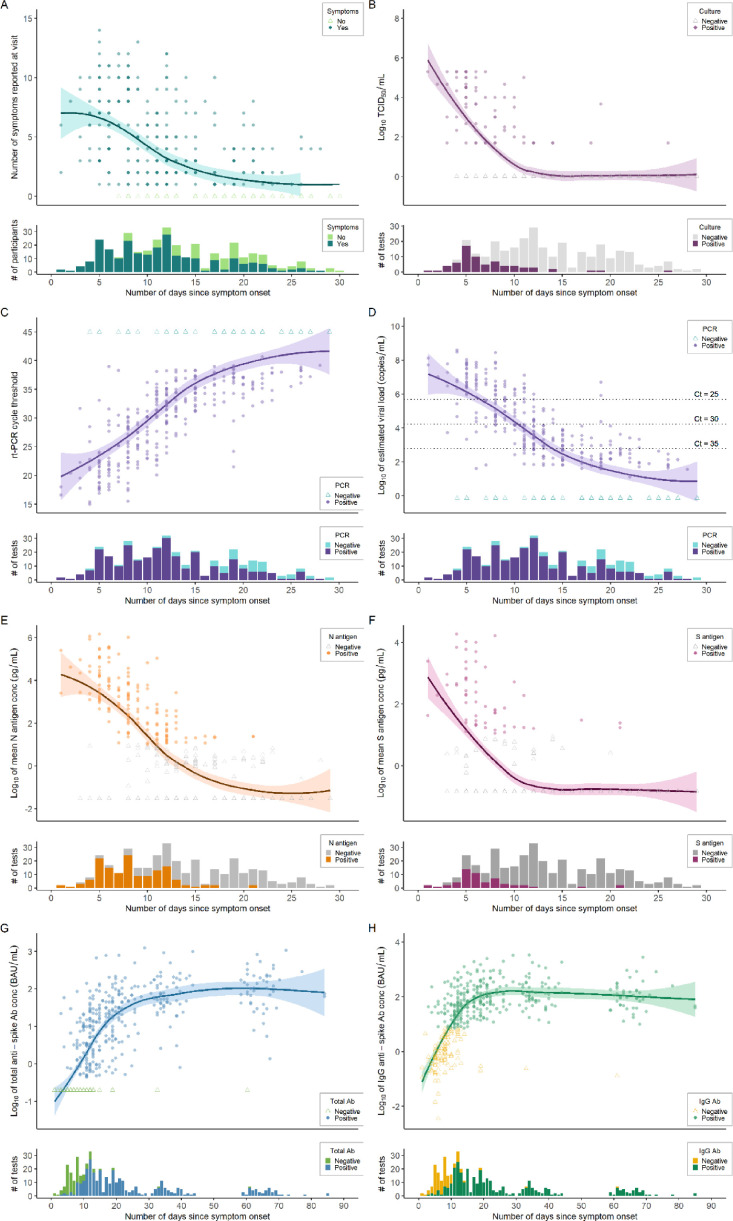
Trajectory of clinical symptoms, replication-competent viral growth, viral load by RT-PCR, nucleocapsid and spike antigen concentrations, and antibody titers, by days since symptom onset. Within each panel, quantitative data are displayed in the top portion and dichotomous positive/negative test results are displayed in the bottom portion, by number of days since date of symptom onset. Average lines represent LOESS curves and shaded regions represent 95% confidence intervals. Darker coloring indicates higher density of observations at that value (e.g., overlapping points lead to darker coloration). A, Total number of COVID-19 symptoms reported by participants at each clinical visit (*n* = 351). B, Viral culture by log_10_ TCID_50_ per mL, with an estimated limit of detection of 2.0 (*n* = 307). C, RT-PCR testing of viral RNA with cycle threshold (Ct) on the vertical axis (*n* = 347). D, Estimated log_10_ of SARS-CoV-2 viral load (copies/mL) from RT-PCR testing (*n* = 347). E, Nucleocapsid (N) antigen log_10_ mean concentration (pg/mL) as measured by a MesoScale Diagnostics assay (*n* = 348). F, Spike (S) antigen log_10_ mean concentration (pg/mL) as measured by a MesoScale Diagnostics assay (*n* = 349). G, Total anti-spike log_10_ mean antibody concentration (BAU/mL) tested by Roche Elecsys assay (*n* = 442), excluding antibody titers obtained after vaccination. H, Anti-spike IgG log_10_ mean antibody concentration (BAU/mL) tested by Abbott AdviseDx assay (*n* = 442), excluding antibody titers obtained after vaccination.

Across the cohort, presence of fever, respiratory symptoms, and loss of taste/smell were not statistically significantly associated with infectiousness during the first 14 days after onset of symptoms (Table 3). Between 6–10 days from symptom onset, presence of N antigen was significantly associated with viral culture positivity [relative risk (RR)=7.61, 95% CI: 3.01–19.22], whereas presence of viral RNA was not statistically significantly associated with culture positivity. Presence of N antigen remained strongly associated with greater risk of infectiousness, despite presence/absence of COVID-19 symptoms. When adjusted for age, sex, comorbidities, and viral variant, presence of N antigen was strongly associated with higher risk of infectiousness within two weeks from symptom onset, both overall and among those with symptoms (overall aRR=7.66, 95% CI: 3.96–14.82) (Table 4). In similar adjusted analyses, RT-PCR positivity was not statistically significantly associated with risk of infectiousness when adjusted for symptoms, but the association was statistically significant among those with fever (aRR=4.12, 95% CI: 1.06–15.91) or respiratory symptoms (aRR=4.25, 95% CI: 1.15–15.66).

**Table 3:** Estimates of relative risk of infectiousness (viral culture positive) based on symptoms (loss of taste/smell, fever, or respiratory), nucleocapsid (N) antigen or RT-PCR test result, and combinations, stratified by days since symptom onset.

	Relative Risk of Infectiousness^a^ (viral culture positive)		
	Days from onset of symptom		
	0 – 5 days (*N* = 110)	6 – 10 days (*N* = 138)	0 – 14 days (*N* = 306)
Symptoms Alone			
Presence of loss of smell/taste	1.07 (0.70–1.63)	0.48 (0.27–0.88)	0.67 (0.45–0.99)
Presence of fever	1.45 (0.84–2.52)	1.09 (0.50–2.42)	1.18 (0.71–1.93)
Presence of respiratory symptoms	2.16 (0.74–6.31)	1.61 (0.55–4.73)	1.48 (0.66–3.29)
Testing Alone			
N Antigen test positive	8.60 (3.50–21.14)	7.61 (3.01–19.22)	7.61 (4.33–13.35)
RT-PCR test positive	—b	3.35 (0.65–17.3)	7.14 (2.09–24.43)
Combined Antigen Test and Symptoms			
N Ag test positive among those with loss of smell/taste	11.57 (3.06–43.78)	7.25 (2.09–25.13)	8.21 (3.76–17.94)
N Ag test positive among those with fever	8.20 (2.89–23.32)	6.89 (2.22–21.38)	6.92 (3.66–13.10)
N Ag test positive among those with respiratory symptoms	6.13 (2.56–14.65)	7.14 (2.88–17.70)	6.67 (3.83–11.64)
Combined RT-PCR Test and Symptoms			
RT-PCR test positive among those with loss of smell/taste	—b	1.97 (0.36–10.74)	3.46 (1.06–11.32)
RT-PCR test positive among those with fever	—b	2.37 (0.41–13.77)	5.90 (1.64–21.22)
RT-PCR test positive among those with respiratory symptoms	—b	3.32 (0.66–16.81)	5.54 (1.67–18.37)

a Results shown are the estimated relative risk with 95% confidence intervals of generalized estimating equations with positive symptoms and/or test results as the predictors of a positive viral culture result.

b Unreliable estimates due to 0 samples that resulted RT-PCR negative and culture positive between 0–5 days.

**Table 4:** Estimates of adjusted relative risk of infectiousness (viral culture positive) based on nucleocapsid (N) antigen or RT-PCR test result between 0–14 days since symptom onset, and stratified by symptoms (loss of taste/smell, fever, or respiratory).

	Adjusted Relative Risk of Infectiousness^a^ (viral culture positive)			
	Regardless of symptoms (*N* = 306)	Persons with loss of smell/taste (*N* = 198)	Persons with fever (*N* = 226)	Persons with respiratory symptoms (*N* = 268)
N Antigen test positive	**7.66 (3.96–14.82)**	**7.33 (3.30–16.32)**	**4.77 (2.56–8.89)**	**5.11 (2.92–8.94)**
RT-PCR test positive	2.74(0.81–9.25)	2.57 (0.75–8.79)	**4.12 (1.06–15.91)**	**4.25 (1.15–15.66)**

This table presents analyses on 306 participant visits–by 95 unique participants–with a complete set of three test results (RT-PCR, N antigen, and culture), within 30 days following symptom onset.

a Results shown are the estimated relative risk with 95% confidence intervals of generalized estimating equations with positive symptoms and/or test results as the predictors of a positive viral culture result. All models were adjusted for age, sex at birth, comorbidities, and SARS-CoV-2 variant.

We used LOESS curves to describe the clinical and diagnostic trajectories by days since symptom onset (Fig. 3). Most participants reported COVID-19 symptoms through 14 days. Replication-competent virus was routinely present in NP swabs through seven days, while only two participants were culture-positive beyond 15 days from symptom onset. One unique individual had trace viral growth (TCID_50_ <100) at 26 days since symptom onset, which was sequenced as viral lineage B.1.1.7. At the visit, the individual was asymptomatic, positive by RT-PCR (Ct=38.3), and N antigen negative.

**Fig. 4:**
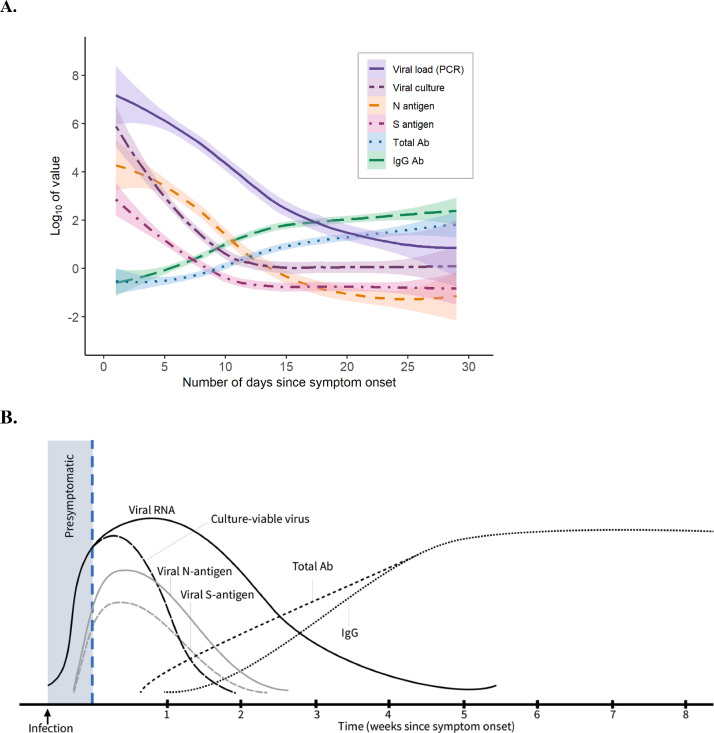
Diagnostic test kinetics and immunological responses in adults with non-severe, symptomatic SARS-CoV-2 infection. Average lines represent LOESS curves and shaded regions represent 95% confidence intervals. The x-axis shows days since symptom onset and the y-axis uses a log transformation. A, Study data with Log_10_ values for measured SARS-CoV-2 viral load, TCID_50_ from viral culture, nucleocapsid (N) antigen and spike (S) antigen mean concentration, and total anti-spike and anti-spike IgG antibody concentrations, by number of days since symptom onset. B, Theoretical model of diagnostic test kinetics and immunological responses, as extrapolated from observed data obtained among unvaccinated adults during acute SARS-CoV-2 infection.

The average peak viral load was 6–8 log_10_ copies/mL (Ct=16–24), which declined to an average viral load of 2–3 log_10_ copies/mL (Ct≈35) after 14 days from symptom onset. While most people remained RT-PCR positive after 14 days, but only 3 participants had a Ct <30 cycles beyond 14 days. Antigenic titers of N and S proteins had lower relative concentrations and faster rate of decline. After 14 days, the vast majority of participants tested negative for both N and S antigens. Humoral immune responses, as measured by total and IgG anti-spike antibodies, appeared within 14 days of symptom onset, plateaued within 30 days, and remained durable between 60–70 days. When including samples collected after COVID-19 vaccination, both total and IgG anti-spike antibody titers were appreciably higher and with a more durable trajectory (Suppl Fig. 2). We aggregated the findings of diagnostic test kinetics, infectivity, and immunological responses to illustrate the **relative** trajectory of results during acute SARS-CoV-2 infection (Fig. 4).

## 4 Discussion

Among ambulatory adults with community-acquired SARS-CoV-2 infection, the period of infectiousness averaged 11 days after onset of symptoms and extended to 15 days for several individuals. During this time period, N antigen testing was the strongest predictor of the risk of infectiousness, and superior to COVID-19 symptom monitoring and molecular RT-PCR testing. RT-PCR and N antigen assay results correlated with viral culture within the first 5 days from symptom onset, while N antigen test results remained significantly associated with infectiousness 6–10 days and across the 0–14 day period after onset of symptoms. Overall, molecular testing by RT-PCR was a highly sensitive test for initial diagnosis, and detection of N antigen in nasal swabs was optimal for determining potential infectiousness during the subsequent isolation period.

Most participants had high SARS-CoV-2 viral load (Ct<25), N antigen positivity, and viral culture growth within 7 days of symptom onset. While most participants’ viral load measurements decreased steadily during the subsequent two weeks of follow-up, a few participants experienced a late viral rebound. Separate plots for persons having initial N antigen positivity, RT-PCR positivity, and viral isolation/culture positivity—all within five days of symptom onset—demonstrated considerable individual heterogeneity of diagnostic test results and trajectories.

Prior models of diagnostic test kinetics, including our own, have relied on limited studies to generate theoretical models. [8,39] Generating empiric longitudinal measurements of viral burden, viral sequence, and immunological responses helped identify the temporal diagnostic test kinetics and heterogeneity among infection-naïve individuals infected with SARS-CoV-2 virus. RT-PCR testing has high diagnostic sensitivity and utility for surveillance of emerging viral variants. Lower relative concentrations of both N and S antigenic titers may contribute to a lower overall diagnostic sensitivity of rapid antigen tests, when compared to RT-PCR. However, the rate of decline for N antigen level tracks more closely with the decrease in replication-competent virus in NP swabs, which may serve as a proxy for potential infectivity.

Other studies have also reported prolonged positive RT-PCR test results for 1–3 months after initial infection. [19,[40], [41], [42], [43], [44]] In our cohort, positive viral cultures almost entirely occurred in samples with a moderate or high viral load (Ct <35). However, low levels of replication-competent virus (below limit of precise TCID_50_ quantification) were isolated in four specimens with a low or undetectable viral loads (one with Ct=35.3; one with Ct=38.3; two were RT-PCR negative) and N antigen test negative. Therefore, our data also suggest that positive RT-PCR specimens with a low viral load (Ct value >≈35 cycles) are unlikely to correlate with recovery of substantial amounts of replication-competent SARS-CoV-2 virus. While TCID_50_ has itself not been empirically linked to risk of transmission, our results indicate that TCID_50_ may serve as a measure of the presence of transmissible virus and NAATs may detect remnant viral RNA beyond the window of infectivity. [[38], [45], [46]]

The genetic diversity of cultured virus within our cohort reflects the local and temporal viral dynamics that occurred during our study period. We observed little viral genetic variation within individual subjects across study time points. Among ten individuals with serial virus isolation and three household contact groups, we observed 0–19 and 0–14 nucleotides changes between viral isolates, respectively. Therefore, the viral genome remained highly conserved over the study time course and across likely transmission events. Thus far, several studies have evaluated the longitudinal diagnostic kinetics among hospitalized adults, [47], [48], [49], [50] but up to now no studies have evaluated synchronous changes in the longitudinal biomarkers, diagnostic kinetics, infectiousness, and immunological responses among ambulatory adults with acute non-severe SARS-CoV-2 infection.

These results, when combined in a comprehensive diagnostic model of acute SARS-CoV-2 infection may help inform testing guidelines and public health practice. The estimated relative risk of culture positivity for a positive N antigen test (versus a negative N antigen test) was robust, regardless of presence of fever or time since symptom onset within 14 days. Therefore, for public health practices, N antigen testing may be the preferred method of testing to determine the recovery of replication-competent virus and potential infectivity between 6–10 days (or 0–14 days) from symptom onset, either with or without the presence of symptoms. Since persons with symptomatic, non-severe SARS-CoV-2 infections may continue shedding viral RNA for weeks or months after the acute infection, without having replication-competent virus, monitoring infection by molecular RT-PCR testing should be discouraged.

This study had several strengths and limitations. While having a larger sample size and more viral diversity would have been ideal, the study was not designed to compare diagnostic results across variant sub-types and recruitment concluded before the emergence of omicron strains. Our analyses excluded several asymptomatic patients with acute SARS-CoV-2, who may be capable of transmitting infection. [51,52] We also limited our nasal swab testing (i.e., RT-PCR, antigen, and culture) to nineteen days after enrollment and therefore could have underestimated estimates of time to negative test, since individuals who never tested negative during follow-up were excluded from the calculation. Strengths of the study were high retention rates for a population of symptomatic adults undergoing repeated invasive sampling procedures and consistency of trained medical assistant performing the swabs over time. The cohort was relatively young and healthy without immune altering conditions, which is more population-representative than studies conducted among elderly or hospitalized populations.

In conclusion, we presented results from a first infection study of ambulatory adults with acute SARS-CoV-2 infection to describe and compare the longitudinal dynamics for viral viability (culture), viral load by RT-PCR, and viral S and N antigen quantification. Importantly, these findings indicate that public health guidance could encourage most persons with acute SARS-CoV-2 infection to remain in contact isolation for at least ten days. They also suggest the use of N antigen testing—since rapid diagnostic tests overwhelmingly target the N antigen—rather than the absence of symptoms or viral RNA, to safely discontinue an isolation period. These results may be used to strengthen infection control measures and reduce SARS-CoV-2 transmission to accelerate ending the COVID-19 pandemic.

## Author contributions

PKD, GG, and MG conceived the study and acquired the funding. RD, PKD, MT, GG, and MG developed the study protocol. RD, JFM, ZM, RP, CW, AG, and PKD acquired the clinical data and specimens. RCI, ER, and DH provided important research infrastructure and sample processing. LH, ALG, MJM, BDG, JLC, ASB, and MJG conducted laboratory testing. MJB, EB, AM, RD, and PKD conducted the statistical analyses. PKD prepared the first manuscript draft. All authors provided critical feedback and approved of the final manuscript.

## Funding

The study was funded by the 10.13039/100000865Bill and Melinda Gates Foundation (#INV-017205).

## Declaration of Competing Interest

The authors declare that they have no known competing financial interests or personal relationships that could have appeared to influence the work reported in this paper.

## Appendix Supplementary materials

Image, application 1
