# Supplementary material for: Duration of viral infectiousness and correlation with symptoms and diagnostic testing in non-hospitalized adults during acute SARS-CoV-2 infection: A longitudinal cohort study
Source: J Clin Virol. 2023 Apr;161:105420. doi: 10.1016/j.jcv.2023.105420 (PMC9981266; doi:10.1016/j.jcv.2023.105420)

**Supplemental Table 1. Average days since enrollment and symptom onset, and specimens tested, by participant visits.**

|  | Visit #1 | Visit #2 | Visit #3 | Visit #4 | Visit #5^a^ | Visit #6^a^ | **Total** |
| --- | --- | --- | --- | --- | --- | --- | --- |
| **Participant attended visit** | 95 | 89 | 83 | 82 | 79 | 79 | **507** |
| Days since enrollment |  |  |  |  |  |  |  |
| Mean | 0.0 | 3.9 | 7.3 | 14.5 | 28.9 | 58.8 |  |
| Median [IQR] | 0 [0, 0] | 4 [3, 5] | 7 [7, 7] | 14 [14, 14] | 28 [28, 29] | 56 [56, 59] |  |
| Min, max | 0, 0 | 3, 6 | 7, 11 | 14, 19 | 27, 34 | 56, 82 |  |
| Days since symptom onset |  |  |  |  |  |  |  |
| Mean | 6.9 | 10.9 | 14.5 | 21.7 | 36.0 | 65.9 |  |
| Median [IQR] | 6 [5, 8] | 11 [9, 13] | 14 [12, 17] | 21 [19, 24] | 35 [34, 39] | 64 [62, 68] |  |
| Min, max | 1, 14 | 5, 18 | 8, 21 | 15, 29 | 29, 44 | 57, 87 |  |
| **Specimens Tested** |  |  |  |  |  |  |  |
| NP for viral load by RT-PCR | 95 | 88 | 82 | 82 | 0 | 2 | **349** |
| NP for viral culture | 83 | 81 | 69 | 74 | 0 | 2 | **309^b^** |
| AN for nucleocapsid (N) antigen | 95 | 88 | 83 | 82 | 0 | 0 | **348** |
| AN for spike (S) antigen | 95 | 89 | 83 | 82 | 0 | 0 | **349** |
| Blood for Total anti-S antibodies | 93 | 89 | 83 | 82 | 79 | 78 | **504** |
| Blood for IgG anti-S antibodies | 93 | 89 | 83 | 82 | 79 | 78 | **504** |
| Viral sequencing (variants) | 46 | 18 | 3 | 3 | 0 | 0 | **81** |

AN=anterior nares; IQR=interquartile range; NP=nasopharyngeal.

^a^Nasal swabs were collected at visit #5 and #6 only for participants who reported any COVID-19-like or respiratory symptoms, and all tested were negative.

**^b^Three samples were excluded from culture analyses presented in Tables 2 and 4 due to missing N antigen result (n=1) or collection beyond 30 days from symptom onset (n=2; see footnote a above).**

**Supplemental Table 2. Diagnostic tests (S antigen, viral culture, N antigen, and RT-PCR for viral RNA) performed on samples collected at the first four visits.** Median time (days) from symptom onset until negative test was determined for each test type, among participants with a negative test result during follow-up (see Figure 1).

| **N=95** | **Study exit before negative test observed^a^**  N (%) | **End of follow-up before negative test observed^b^**  N (%) | **Observed test-negatives**  N (%) | **Days since symptom onset of observed test-negatives**  Median [IQR] |
| --- | --- | --- | --- | --- |
| S antigen | 4 (4) | 0 | 91 (96) | 9 [5] |
| Culture | 7 (7) | 0 | 88 (93) | 11 [4] |
| N antigen | 6 (6) | 1 (1) | 88 (93) | 13 [6] |
| RT-PCR | 12 (13) | 45 (47) | 38 (40) | >19^c^ |

IQR=interquartile range.

^a^Of 95 sampled participants, some exited before a negative test could be observed (treated as non-informative and excluded from the median analysis).

^b^Other participants attended study visits but were positive at time of last collected nasal swabs for diagnostic testing (V4). If these individuals could have been observed longer, time to test negative is expected to be greater than the median estimated using observed test-negatives.

^c^Median [IQR] for RT-PCR could not be precisely estimated because more than half of participants were positive at all available sample times (>2-4 weeks after onset of symptoms).

**Supplemental Figure 1. Consort diagram of study recruitment and enrollment.**


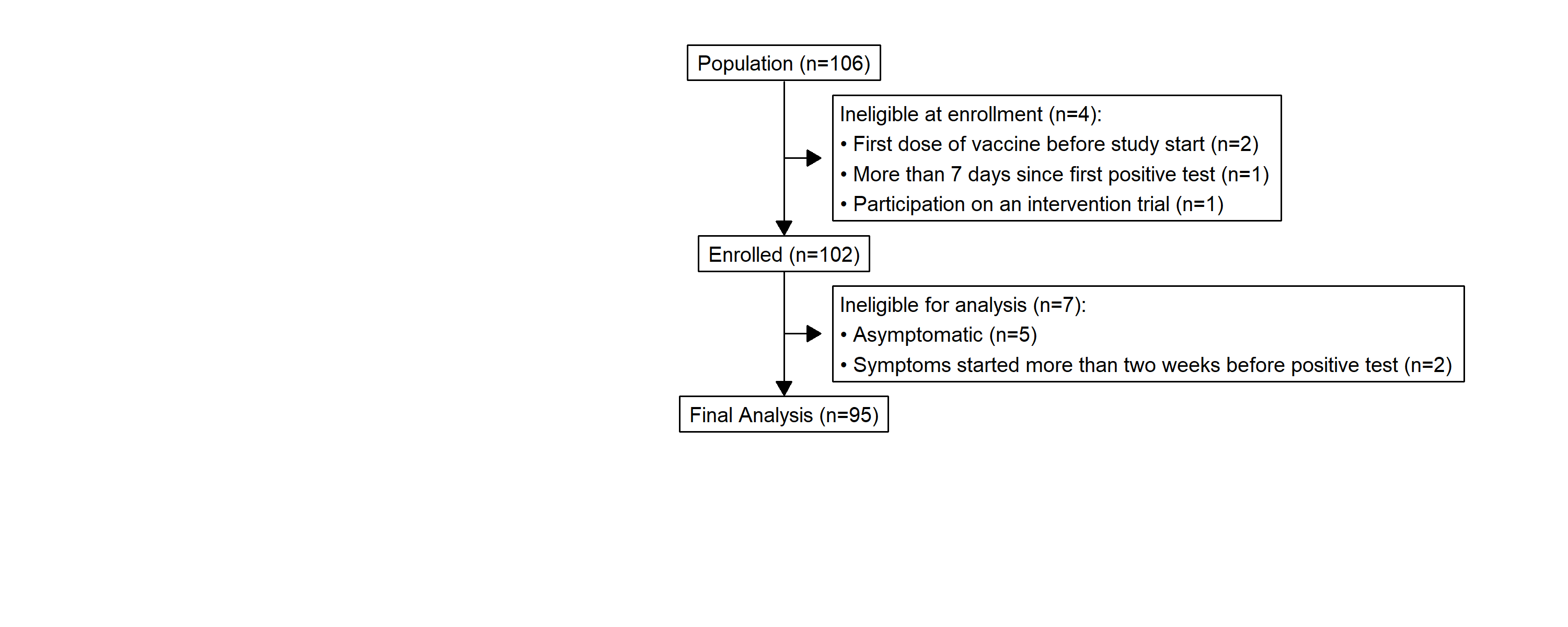


Among COVID-positive longitudinal study participants, of the 106 participants recruited, four were ineligible. Two people had received COVID-19 vaccination, one tested positive >7 days before enrollment, and one participated in a clinical trial). Seven participants were subsequently excluded from analyses: five because they remained asymptomatic, and two who were symptomatic >2 weeks prior to diagnostic testing.

**Supplemental Figure 2. Trajectory of Total and IgG antibody titers, by days since symptom onset and with the inclusion of individuals who received COVID-19 vaccination during the study period.** Average lines represent LOESS curves and shaded regions represent 95% confidence intervals. Color indicates qualitative result, and shape indicates vaccination status at the time of sample collection. **A,** Total anti-spike log_10_ mean antibody concentration (BAU/mL) tested by Roche Cobas e411 system (n=504). **B,** Anti-spike IgG log_10_ mean antibody concentration (BAU/mL) tested by Abbott AdviseDx platform (n=504).


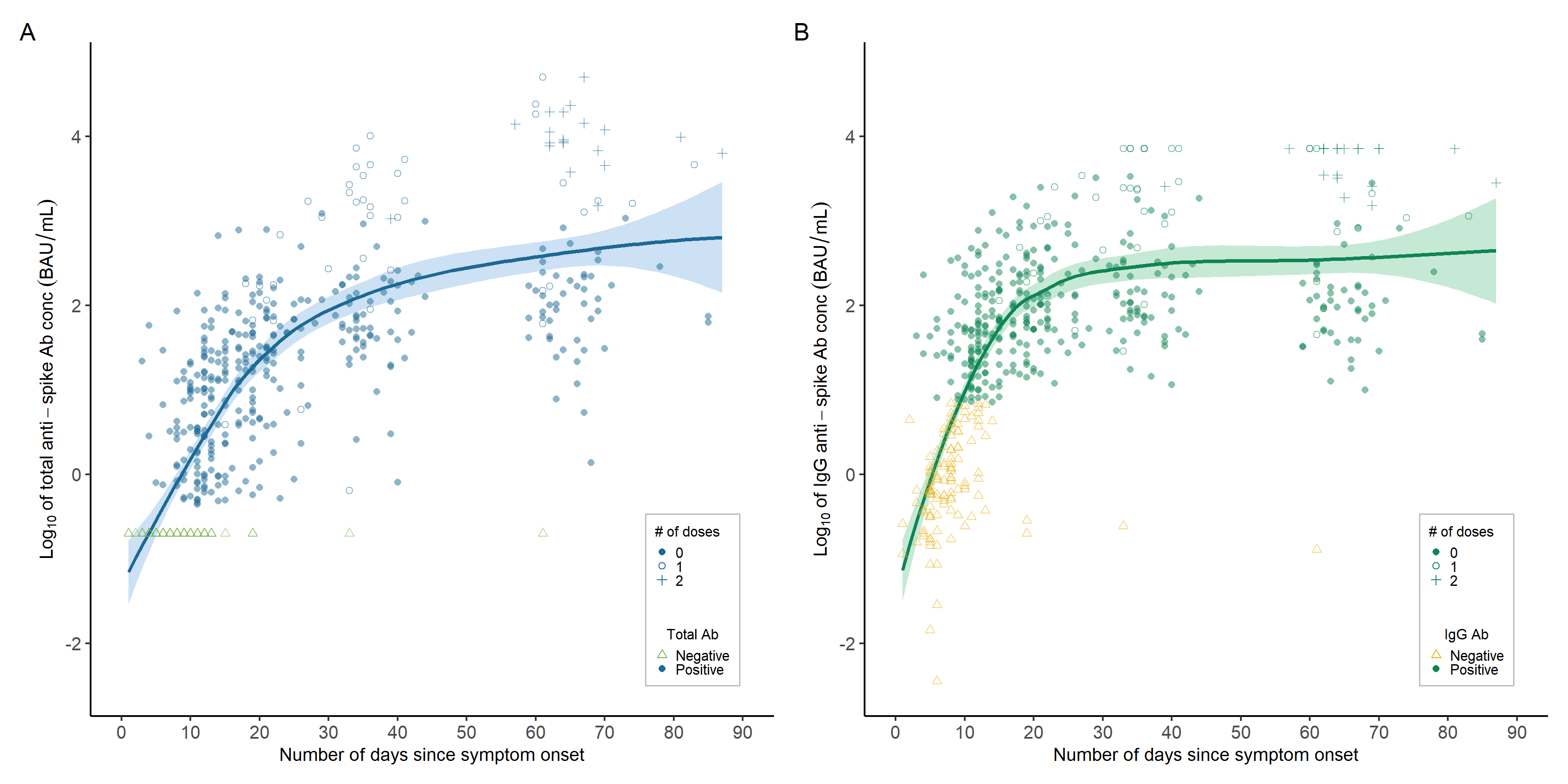


**Supplemental Figure 3. Individual trajectories for diagnostic test kinetics and infectivity during acute SARS-CoV-2 infection.** Spaghetti plots show serial testing with one connected line for each individual participant indicating change in results over time. Darkened lines highlight participants who displayed apparent viral “rebound”. The x-axis shows days since symptom onset, and RT-PCR Ct value is indicated on y-axis. Viral culture result (positive/negative) is indicated by color (orange=viral growth; teal=no viral growth; purple=not cultured); matched N antigen test result is indicated by fill (hollow=negative, solid=positive). Horizontal lines present RT-PCR limit of detection (Ct=42.0) and Ct=35.0 for reference. **A,** All participants. **B,** Participants with positive N antigen test within five days of symptom onset. **C,** Participants with positive RT-PCR test within five days of symptom onset. **D,** Participants with positive viral culture result within five days of symptom onset.

***[Note: Panel figure on next page]***


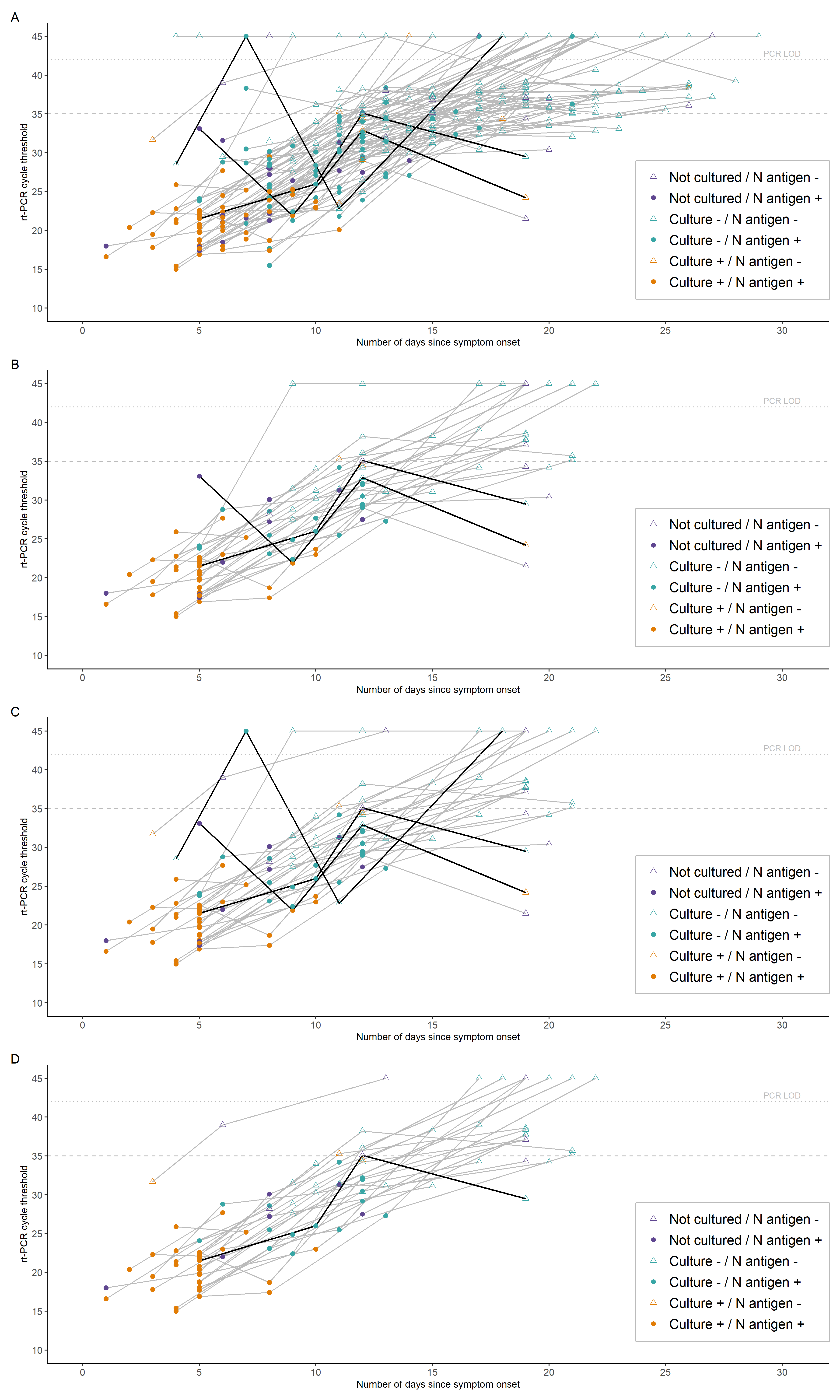

Supplement: Supplementary file 1 [file mmc1.docx]
